# Is a new generation of mycotoxin clay adsorbents safe in a pig’s diet?

**DOI:** 10.1186/s40813-022-00275-w

**Published:** 2022-07-04

**Authors:** Pavel Horky, Pavel Nevrka, Tomas Kopec, Iqra Bano, Misa Skoric, Jiri Skladanka, Sylvie Skalickova

**Affiliations:** 1 Department of Animal Nutrition and Forage Production, Faculty of AgriSciences, Mendel University in Brno, Zemedelska 1, 613 00 Brno, Czech Republic; 2 Department of Animal Breeding, Faculty of AgriSciences, Mendel University in Brno, Zemedelska 1, 613 00 Brno, Czech Republic; 3 Department of Pathological Morphology and Parasitology, Faculty of Veterinary Medicine, University of Veterinary and Pharmaceutical Sciences Brno, Palackeho trida 1946/1, Brno, 61200 Czech Republic; 4 Department of Physiology and Biochemistry, Faculty of Bio-Sciences, Shaheed Benazir Bhutto University of Veterinary and Animal Sciences, Sakrand, 67210 Sindh Pakistan

**Keywords:** Growth performance, Health status, Histopathology, Bentonite

## Abstract

**Background:**

Bentonites, as a clay mineral, serve in pig farms as adsorbents of toxic substances. They are mainly used to reduce the negative impact of mycotoxins to maintain the performance and health status of animals. The new genotypes of pigs are highly sensitive to a range of antinutrients, including mycotoxins. Currently, attention is focused on more effective adsorbents of mycotoxins with a higher adsorption capacity. Such materials are in great demand among feed manufacturers. However, there is a concern that these new materials may also adsorb too many essential nutrients and decrease animal performance. The aim of the experiment was to evaluate the effect of the new generation of purified bentonites on the efficiency and health status of the pigs.

**Results:**

Forty-eight slaughtered pigs with an average weight of 31.2 ± 2.6 kg were included in the experiment. The pigs were divided into two groups (2 × 24 pigs). Pigs were slaughtered at an average weight of 66.3 ± 5.2. The first group had a diet without clay (control—C). The second group (treatment—T) was fed a diet with a clay additive (purified bentonite) of 1.5 kg/t. Animals were fed the experimental diet for 35 days. In group T, a higher daily weight gain (by 4.8%) and feed intake (by 2.9%) was observed while the feed conversion decreased by 1.9%. There were no significant differences between the groups of pigs during observation in the evaluation of hematological, biochemical parameters of the blood. Morpho-pathological analysis of the jejunum showed similar signs of moderate lymphoplasmacytic infiltrate in the mucosa in the groups C and T, contained similar number of goblet cells.

**Conclusion:**

Taken together, the addition of the new generation of bentonite clays did not negatively influence the health status and the performance of pigs.

## Background

Bentonite is a mineral authorized in Europe as a feed additive for reducing mycotoxin contamination in feed (EU Reg. No. 1060/2013) [1]. Clays are crystalline, hydrated aluminosilicate molecules composed of alkali and alkaline earth cations along with small amounts of various other elements [2]. Approximately 50 years ago, modern scientists rediscovered the medical purposes of clay minerals, even though eating clay to promote internal healing had been used for hundreds of years by animals and indigenous cultures around the planet [3]. Non-domesticated animals search for clay deposits and consume these minerals for detoxification of the body from anti-nutritional compounds contained in their diet or for alleviation of gastrointestinal disorders [4].

Clays have been a standard ingredient in pig feed mixtures for several decades. In particular, bentonites, kaolins and zeolites hold the major share of the global market [5]. Due to their high adsorption capacity, they are widely used in animal nutrition. Clays can bind a large number of economically significant mycotoxins and they are useful in the balancing of heavy metals or dioxins in the gastrointestinal tract. The current trend is to use clay minerals to prevent diarrheal infections in weaned piglets [6]. Other studies report an antiparasitic effect and an overall detoxification effect on the animal organism. Various bentonites used in animal nutrition differ from each other in several aspects. It has been shown that bentonites are diverse in active surface, porosity and hydrophobicity. These properties are related to the binding of biological molecules and mineral compounds. While bentonite can absorb many organic and inorganic materials in an animal's gastrointestinal tract, it is reported not to affect mineral metabolism [7]. One of the main aspects is the size of the particles, which is crucial for increasing the adsorption capacity [8]. There is a general belief in the scientific community that clays with high adsorption capacity can balance essential nutrients in addition to antinutrients.

Adding clays to pig feed mixtures can potentially lead to a decrease in animal performance or damage to health. High adsorption capacity is characteristic particularly for bentonites. This property is mainly due to a very fine structure and a large adsorbent surface [9]. According to the European Food Safety Authority (EFSA) the safe level of algae interspaced bentonite is 0.125 kg/ton/complete feed for pigs. No negative effects on performance or genotoxic effects were observed at this level. The additive is not an irritant to the skin or eyes and it is considered to have low inhalation toxicity [10]. When assessing the components bentonite and sepiolite, the maximum safe level of these clays was set by EFSA at 20 kg/ton /complete feed. This level is set to be safe for all livestock species [11].

Bentonites can adsorb large organic molecules, polymer substances, complex ions, enzymes or ammonia. Thus, mineral nutrients such as zinc, manganese, selenium, cobalt, etc. can also be bound, with negative effects. Another potential risk of clay adsorbents is their toxicity, which is linked to the extraction site. If clays are obtained with natural or anthropogenic toxic compounds, such a product becomes potentially dangerous to the health of the pigs [12].

Recently, there has been a trend of a steady increase in the adsorption capacity of bentonites used in animal nutrition. This effect can be achieved via advanced technological processes involved in grinding, purification and filtration. One goal of the scientific community should be to verify the safety of these processed bentonites for the organism health of animals. [13]. Purified bentonites are characterized by a higher absorption capacity due to the large area and porosity of the material, compared to conventional forms. Thus, there may be concerns that their high binding capacity may cause the binding of essential nutrients. The aim of this work was to verify whether bentonite adsorbents of mycotoxins and other antinutrients based on a new generation of purified bentonites have a negative effect on the performance and health status of fattening pigs.

## Results

### Growth performance

In assessing the pig’s growth performance, no significant differences were observed between each stage of the experiment. The group with the addition of clay adsorbent showed a higher feed intake by 0.06 kg/day during the entire experiment. The feed conversion was lower by 0.04 kg and the daily gain was higher by 0.05 kg/day also in group T. The performance of the individual indicators during the experiment can be seen in Tables 1 and 2.

**Table 1:** Growth performance of pigs

Feed efficiency traits	Group		*p* value
	C	T	
1–14 days			
Feed intake (kg/day)	1.75 ± 0.14	1.83 ± 0.07	0.4124
Feed conversion (kg/kg)	2.01 ± 0.04	1.95 ± 0.08	0.3992
Weight gain (kg/day)	0.87 ± 0.06	0.94 ± 0.04	0.4701
15–28 days			
Feed intake (kg/day)	2.22 ± 0.08	2.24 ± 0.16	0.3412
Feed conversion (kg/kg)	2.06 ± 0.07	2.06 ± 0.07	0.8601
Weight gain (kg/day)	1.07 ± 0.05	1.09 ± 0.09	0.5560
29–35 days			
Feed intake (kg/day)	2.45 ± 0.15	2.55 ± 0.28	0.2621
Feed conversion (kg/kg)	2.42 ± 0.20	2.36 ± 0.24	0.2227
Weight gain (kg/day)	1.01 ± 0.09	1.08 ± 0.10	0.6877
For the whole experiment			
Feed intake (kg/day)	2.08 ± 0.09	2.14 ± 0.14	0.5302
Feed conversion (kg/kg)	2.12 ± 0.07	2.08 ± 0.08	0.4854
Weight gain (kg/day)	0.98 ± 0.04	1.03 ± 0.05	0.3845

**Table 2:** Pigs’ weight (kg) during the experiment

Time period	Group		
	C	T	*p* value
0 day	31.0 ± 2.6	31.3 ± 2.7	0.7053
14 day	43.2 ± 3.7	44.5 ± 4.0	0.2703
28 day	58.3 ± 5.0	59.7 ± 4.8	0.3239
35 day	65.3 ± 5.2	67.3 ± 5.2	0.2045

### Liver and kidneys weight

Liver and kidney weight was related to the body weight of pigs. From Table 3 it is evident that group T did not significantly differ from group C. These results indicate that mycotoxin adsorbent T did not affect body weight and the development of livers and kidneys.

**Table 3:** Liver and kidneys percentage (%) of body weight

Time period (kg)	Group		
	C	T	*p* value
Liver (%)	2.02 ± 0.11	2.14 ± 0.15	0.1732
Kidneys (%)	0.36 ± 0.02	0.41 ± 0.05	0.0996

### Haematological, biochemical and antioxidant parameters of pig’s blood

There were no significant differences between the control group and the treated group in assessing biochemical hematological and antioxidant parameters. The individual parameters corresponded to the physiological levels for the animal species. The results we can see in Table 4.

**Table 4:** Antioxidant status, haematological and biochemical parameters

Time period (kg)	Group		*p* value
	C	T	
GSH (μmol/L)	6.28 ± 2.43	8.88 ± 2.27	0.1334
GSSG (μmol/L)	8.85 ± 3.78	11.39 ± 3.85	0.3572
GSH/GSSG	1.02 ± 0.78	0.72 ± 0.22	0.4769
MDA intestine (nmol/mg)	41.7 ± 8.1	49.5 ± 3.7	0.0585
MDA blood (nmol/mL)	4.15 ± 1.02	4.48 ± 0.61	0.5071
GPx (μkat/L)	445 ± 115	339 ± 47	0.4292
Albumin (g/L)	27.5 ± 2.5	26.6 ± 4.0	0.6737
Hemoglobin (g/L)	135 ± 12	134 ± 15	0.8147
Leukocytes (g/L)	5.70 ± 1.23	11.87 ± 6.09	0.3355
Hematocrit (L/L)	0.35 ± 0.02	0.36 ± 0.03	0.7386
Platelets (g/L)	444 ± 80	439 ± 60	0.8980
Erytrocytes (t/L)	6.01 ± 0.47	5.93 ± 0.55	0.7895
Albumin (g/L)	29.8 ± 2.8	30.2 ± 3.1	0.2699
AST (μkat/L)	0.54 ± 0.03	0.89 ± 0.32	0.1675
GGT (μkat/L)	0.34 ± 0.09	0.36 ± 0.09	0.7681
Creatinine (μmol/L)	102 ± 8	101 ± 15	0.9446
Urea (mmol/L)	4.22 ± 1.01	5.45 ± 1.60	0.2246
Glucose (mmol/L)	5.43 ± 0.50	4.90 ± 0.74	0.2071
SDH (nmol/L)	0.017 ± 0.002	0.016 ± 0.002	0.2071

GSH, reduced glutathione in the blood; GSSG, oxidized glutathione in the blood; GSH/GSSG, a ratio of reduced glutathione to oxidized glutathione; MDA, malondialdehyde GPx –glutathione peroxidase; AST, aspartate transaminase; GGT, Gamma-glutamyl transferase; CREA, creatinine; GLUC, glucose; SDH, sorbitol dehydrogenase

### Morpho-pathological analysis of the middle jejunum and liver

**Fig. 1:**
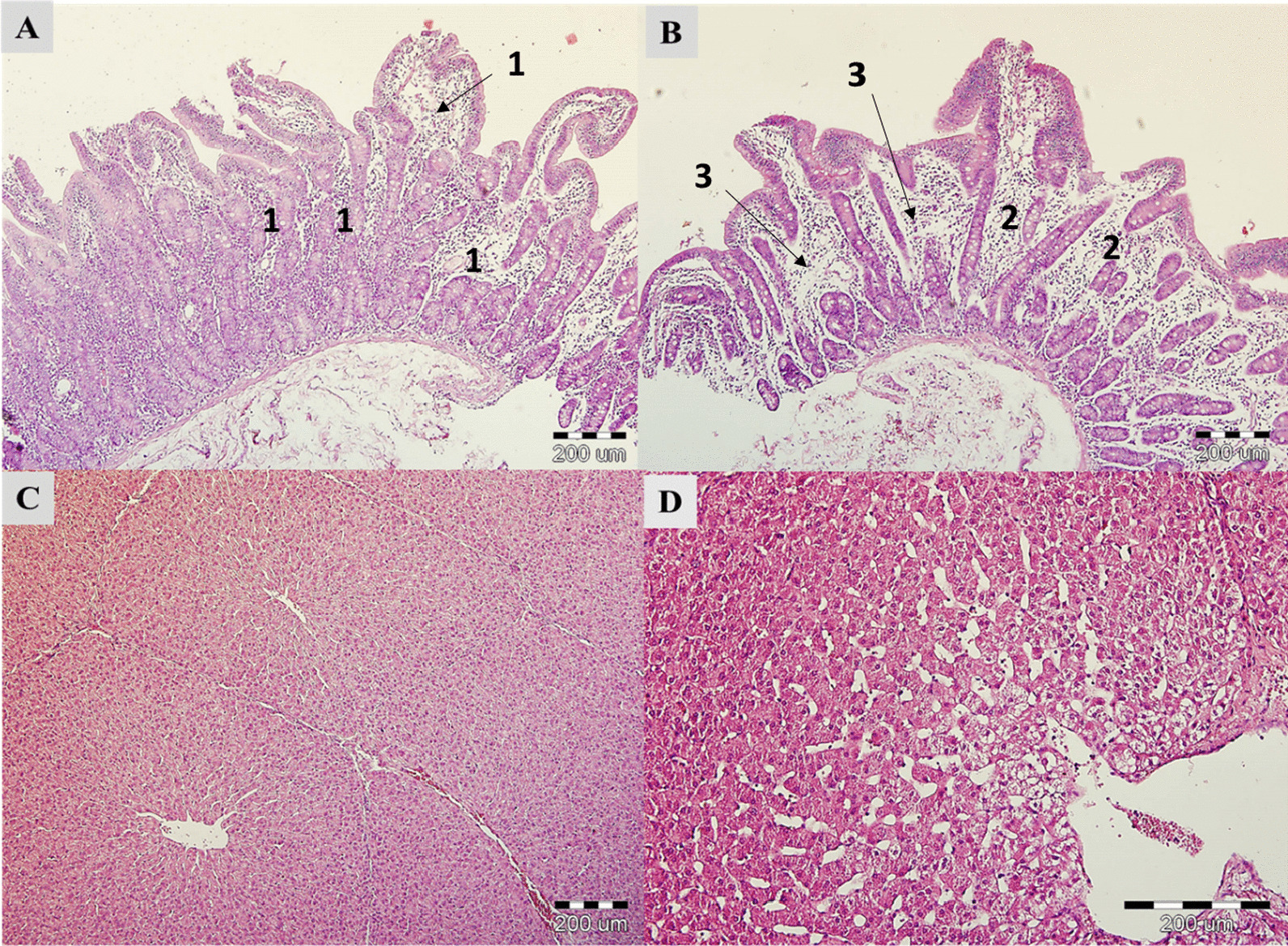
Morpho-pathological analysis of the middle jejunum and liver. Middle jejunum: control group (**A**); treated group (**B**); liver: control group (**C**); treated group (**D**)

In group C, a lymphoplasmic inflammatory infiltrate in the mucosa, an abundance of eosinophils and isolated macrophages were observed. Lymphangiectasia of some villi and lymphatic vessels in the submucosal layer were found in some of the biopsies. The average number of goblet cells was 49 (counted in the HPF). Figure 1A shows a moderate lymphoplasmacytic infiltrate and eosinophilic infiltrate in the lamina propria (No. 1). Group T (Fig. 1B) had a predominantly moderate focal lymphoplasmacytic inflammatory infiltrate in the intestinal mucosa (No. 2), abundant eosinophils, with mild edema of lamina propria (No. 3), lymphangiectasia of some villi, mean goblet cell count (43/ HPF). In the analysis of the liver of animals from C and the T groups, no pathomorphological changes were found. The normal liver parenchyma was free of inflammatory and degenerative changes or other pathology (Fig. 1C, D).

## Discussion

Some authors are concerned that the use of new materials with higher adsorption capacity, such as functionalized bentonites by acid-activation, will lead to a decrease in animal performance [1]. Other studies suggest that bentonites may be characterized by genotoxicity, DNA damage [14], or increased antioxidant stress [15]. In this experiment, several markers have been used for the evaluation of health status and growth performance of pigs. The aim of this research was to evaluate the impact of purified bentonites on swine health and performance. No significant differences were found between treated and control group in term of feed intake, weight gain and feed conversion. According to Subramanian and Kim [16], higher growth performance can be explained by an increase in the digestibility of individual nutrients. In general, clays reduce the rate of passage of feed through the digestive tract, which increases the time for the digestive process itself. Nutrient digestibility depends on the age of the pigs. With increasing age, the retention of nutrients in the gastrointestinal tract (GIT) increases and its digestibility decreases. The average retention time in the GIT of pigs in finishing pigs (75 kg BW) is 37 h. For sows, this time is extended to 81 h [17]. Thus, it cannot be stated that the slow passage of GIT feed will affect nutrient uptake. A number of factors affect nutrient adsorption: feed composition, feeding technology, fiber content, animal age, health status [18].

The improved growth performance of fattening pigs after the addition of silicate clay was also investigated in another study by Li et al. The authors explained this effect by the fact that clays can improve the integrity of the intestinal wall and positively influence the intestinal microbiome [19]. After the addition of clays (3 kg/ton), a higher increment (by 8.5%) and a lower conversion (by 5.4%) were observed in weaned piglets [20]. In other research, an even greater increase of 45% and a lower conversion of 16.9% were observed with administering 1% kaolin to the pigs' diet [21]. Analogously, Li and Kim [19] reported that growing pigs fed a diet supplemented with 0.5% sericite had 6.6% higher weight gain and a 5.1% better feed conversion ratio compared with pigs fed a diet without supplements.

In our experiment, we found a similar trend. For group T, the daily weight increment was insignificantly higher by 5.1% and the feed conversion improved by 1.9% over the duration of the experiment. Other studies also indicate that the addition of clay (montmorillonite) to the diet of weaned pigs at doses of 0–5% did not affect the animal’s performance. In contrast, feed intake decreased linearly with increasing doses [22].

In our follow-up, the addition of purified bentonites had an inconclusive but positive effect on feed intake (an increase of 2.9%). The results of studies on the effect of clay substances on the diet of animals are inconsistent and the values vary from one observation to another. According to Alexopoulos et al. [23] this effect is due to the age of the animals. A higher increment of 14.3% was observed with young, weaned pigs (up to 25 kg bw). Meanwhile, from 25 to 110 kg, an increase in live weight was observed after the addition of clay additive by 5.3%. These results are also consistent with our study, where the gain was higher by 5.1% at a weight interval of 31.2–76.1 kg.

A dose of 1 or 2% bentonite in the diet of weaned piglets did not significantly affect the performance of the animals as in our study [24]. From these results, it can be concluded that bentonite does not have a negative impact on the performance of fattening pigs. During the assessment of the health status of the pigs included in the experiment, no significant difference was observed within our monitoring of hematological and biochemical parameters. A similar trend was observed in research by Holanda and Kim [25]. They found that AST, ALT, ALP were in the physiological range. The issue under discussion is the ability of clays to balance mineral substances. However, for calcium, sodium, potassium and chloride, no difference was observed in the blood of fattened pigs after the addition of clay adsorbents [25]. In an experiment conducted by colleagues Bederska-Lojewska and Pieszka [26], researchers added kaolin clay (100%) to the diet of fattening pigs at a dose of 6 kg/ton of feed. Hematocrit in the group with the addition of clay was significantly higher (39.65%). In our experiment, there were no observable differences between the groups. The hematocrit in group T was 36%, which corresponds to the physiological values for pigs. In addition, the above-mentioned researchers observed a clear reduction in erythrocytes in the group with the addition of clay (6.35 t/l). In our experiment, the erythrocyte value was 5.93 t/l. All values are within the physiological range. It must be noted the authors examined 4-fold higher feed dose of bentonites compared to our study. Thus, the results of these two experiments cannot be compared objectively.

In assessing the biochemical parameters in the pigs’ blood, an evidential decrease in urea was observed in the experimental group of 1.98 mmol/L. We monitored the values of urea in the blood of pigs to a higher _____ in the control group—4.22 mmol/L, the experimental group had 5.45 mmol/l. The differences between our values and the values of Bederska-Lojewska and Pieszka (2019) are likely to be due to the different protein representations in the pigs’ diet. Further evidence-based changes were observed in AST. This enzyme was shown to be higher in the group of animals supplemented with clays [26]. The results mentioned above are different from our follow-up. In our experiment, no changes in hematological or biochemical parameters of blood were observed. In contrast, another research team, Trckova et al. [24], carried out the addition of 1 or 2% bentonite to the diet of weaned piglets.

Trckova et al. [24] have been observed the leukocyte count in the kaolin enriched experimental group was significantly reduced by 19%. In our experiment, a leukocyte counts both, in group C and group T were observed in physiological range. However, the measured values varied within the physiological range. The health status of the small intestine was also assessed in various studies. After the addition of 1% bentonites, no significant differences between the control and the experimental group were observed. Specifically, pathomorphometric evaluation of the length and depth of intestinal crypts and possible inflammatory processes did not show significant changes in the treated group compared to control.

After overcoming mild infection, the following late-phase inflammatory responses were observed: minor destructions and desquamations of the intestinal epithelium with enterocyte shedding into the gut lumen, hyperaemic areas and areas with intestinal crypt hyperplasia. No marked differences between control and bentonite piglets were observed.

The authors Trckova et al. stated that mild signs of inflammation were observed in the control group and experimental group fed by kaolin enriched feed [24]. These results fully correlate with our findings. However, vetween groups C and T, no significant difference in the integrity of the intestine was found. A weak inflammatory infiltrate was monitored in both groups. In another experiment, bentonite was added to the pigs' diet in a dose of 1 or 2%. Even in this case, bentonite did not have a significant negative or positive effect on the histopathological evaluation of the small intestine [27]. Many scientific teams did not pay attention to the evaluation of the morpho-pathological analysis of the liver after adding bentonites to the diet of pigs. In our follow-up, both groups had normal liver parenchyma. Thus, it can be assumed that purified clays did not have any toxic effect on the liver. The conclusions of the study indicate that purified bentonites do not significantly affect the performance or health status of fattening pigs. It is known that this category of animals is not among the most sensitive. In order to complement the breadth of research, it would be beneficial to experimentally verify the effect of purified bentonites of the new generation on other categories of pigs that may be more sensitive to nutritional supplements (piglets, pregnant and lactating sows).

## Conclusion

Pigs have become sensitive to the negative effects of the environment. The goal of nutrition experts is to develop new materials that can eliminate the effects of toxic substances on the body (mycotoxins, dioxins, enterotoxins, etc.). Thereby, evolutionary materials appear, which are characterized by a larger surface area and a higher adsorption capacity. Due to high adsorption capacity and large surface area, there are concerns about their enhanced adsorption of essential nutrients and decrease the performance of pigs. In our experiment, no adverse effect on the performance of fattened pigs, or biochemical and hematological parameters of blood was observed. There was also no difference between the control group and the experimental group of pigs during assessment the morpho-pathology of the small intestine and liver. Based on our results, we can conclude that purified bentonites do not pose a risk to fattening pigs. However, it would be advisable to test the results experimentally on more sensitive categories of pigs.

## Material and methods

The study was conducted according to the guidelines of the Declaration of Helsinki, and approved by the Institutional Review Board (or Ethics Committee) of the Expert Commission for Ensuring the Welfare of Experimental Animals of Mendel University in Brno (protocol code 16OZ27083/2014-17214 and date of approval 20 May 2019). A total of 48 experimental pigs—castrated males (DanBred) with an average weight of 31.2 ± 2.6 kg were housed in 12 identical pens measuring 2.43 × 1.46 m. The animals were exposed to artificial lighting sources with a light intensity of 45 lx in the mode 12 h-light and 12-h dark cycle. According to the pre-fattening pigs' requirements, the microclimatic conditions of pigs' housing were maintained by an artificial computer-controlled ventilation system. The basic microclimatic standards were maintained following the requirements of the Act on the protection of animals against cruelty (No. 246/1992 Coll.). The composition and nutritional values of the feed rations are shown in Tables 5 and 6. The diet was compiled according to the Nutritional Requirements for DanBred Pigs.

**Table 5:** Ingredient composition of the experimental diet for pigs (%)

Ingredient	%
Wheat	48.00
Maize	10.00
Barley	10.95
Pea	4.00
Wheat bran	2.00
Wheat flour	5.00
Extracted soybean meal	10.60
Extracted rapeseed groat	5.00
Animal fat	0.50
L-Lysine HCl 98	0.26
L-Threonine 98	0.14
DL Methionine 99	0.04
L-Tryptophan 20	0.14
Calcium carbonate (ground limestone)	1.04
Feeding salt	0.47
Monocalcium phosphate	0.87
Mineral premix^A^	1.00

^A^Provided per kg of complete diet: Vitamin A, 5000 IU; Vitamin D3, 800 IU; Vitamin E, 30 IU; Vitamin K3, 1.0 mg; Biotin, 0.05 mg; Folic acid, 0.3 mg; Niacin, 10 mg; D-pantothenic acid, 10 mg; Riboflavin, 3.6 mg; Thiamine, 1.0 mg; Pyridoxin, 1.5 mg; Choline, 800 mg; Zn (ZnSO_4_), 120 mg; Fe (FeSO_4_), 125 mg; Cu (CuSO4.5H2O), 15 mg/kg; Mn (MnSO_4_.H_2_O), 10 mg/kg; I (KI), 0.15 mg; Se (Na_2_SeO_3_), 0.2 mg

**Table 6:** Chemical composition of the feed rations (dry matter)

Indicator	Feed rations
Metabolizable energy (MEp), MJ	13.10
Dry matter (%)	88.87
Protein (%)	16.41
Starch (%)	39.61
Fat (%)	2.47
Fiber (%)	3.52
Ash (%)	5.56
Amino acids, (g)	
Lysin	10.64
Methionine	3.37
Methionine + cysteine	7.18
Threonine	7.74
Tryptophan	3.70
Arginine	11.23
Histidine	4.85
Isoleucine	7.71
Leucine	13.93
Phenylalanine	8.88
Valine	9.10
Tyrosine	6.26

All experimental animals were provided ad libitum access to feed and drinking water. In addition, the unconsumed feed mixture was monitored.

**Fig. 2:**
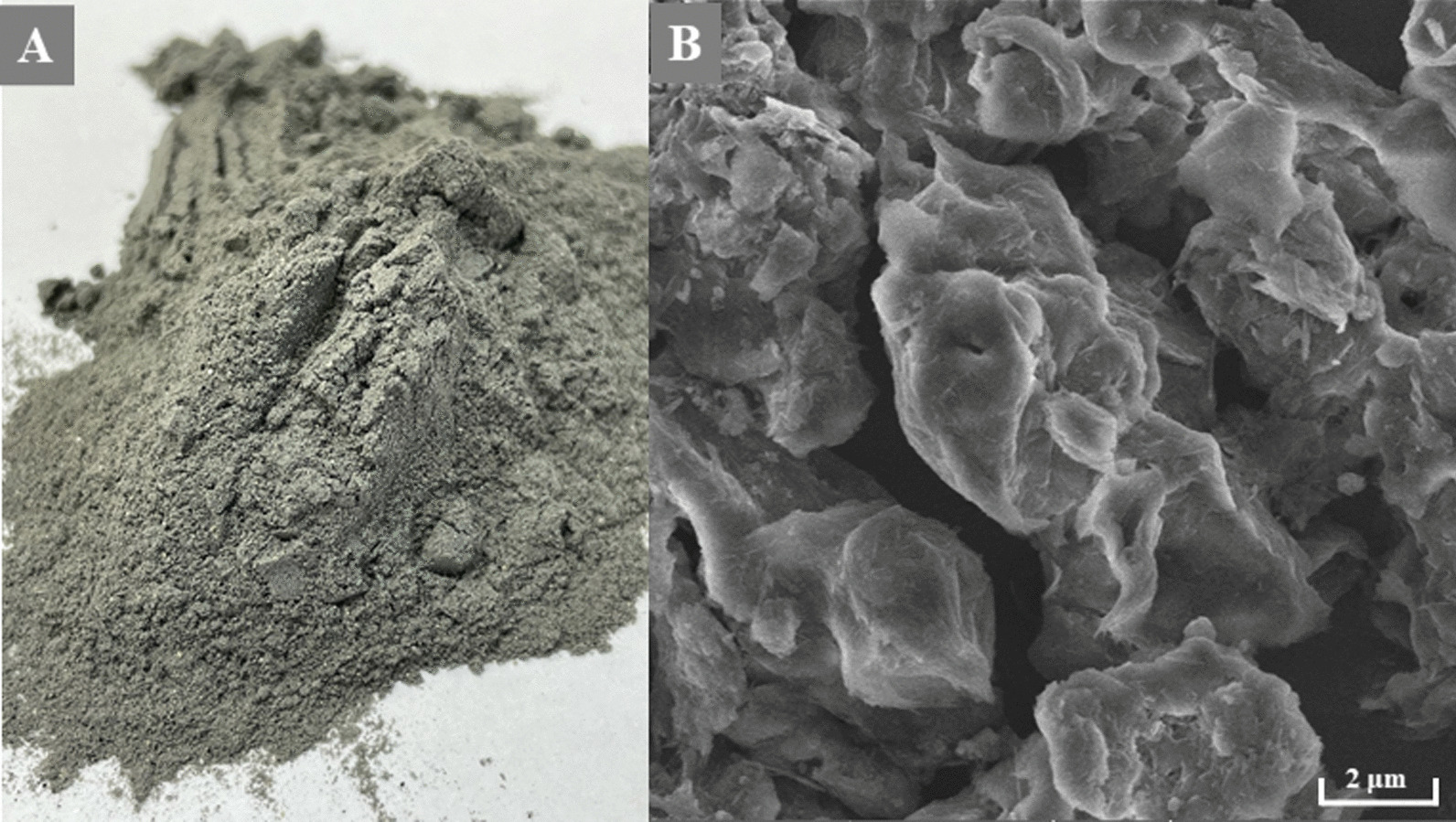
Structure of purified and activated bentonite at macroscopic magnification (**A**) and magnification SEM, (**B**)

A total of 48 pigs was included in the experiment at an average weight of 31.2 ± 2.6 kg. Animals were fed the experimental diets for 35 days and were divided into two groups of 24 animals (6 pens × 4 pigs). The first group had a diet without bentonite addition (control—C). The second group (treatment—T) was fed a diet with a purified bentonite of 1.5 kg/ton (based on a product Fortisorb Premium, Addicoo group, s.r.o., Czech Republic; surface area 300 m^2^, average particle size 30 µm, low polarity, pH does not affect adsorption efficiency due to a high cation exchange capacity; dosing was chosen according to the manual) and served to test the mycotoxin adsorbent's potential adverse effect. Clay adsorbent was based on purified and activated bentonite (Fig. 2A, B).

The weight of the animals was monitored at regular weekly intervals. Average daily feed intake and feed conversion were monitored for the periods: 1–14 days; 15–28 days; 29–35 days. Six pigs from each group were slaughtered in the final weight range of 54.6–76.1 kg (average weight 66.3 ± 5.2). The animals were left to rest and fast for about two hours before slaughtering. Then, they were slaughtered by electrical stunning (350 V, 4 A) and exsanguination. In each slaughtered pig, kidney and liver weight were determined as percentages of the pigs' live weight. Blood samples were collected via the jugular vein into EDTA tubes and 10 mL heparinized vacuum, and then aliquots were centrifuged at 3000×g for 10 to collect plasma which was frozen at − 20 °C until analysis. Liver and intestine samples were collected, washed in PBS buffer, and placed in 10% formalin until evaluation. Middle jejunum (3.5 m distal from duodenum) samples were collected and frozen at − 20 °C until analysis.

### Scanning electron microscopy

For documentation of the activated bentonite structure (Fig. 2B), a scanning electron microscope MIRA3 LMU (Tescan, Czech Republic) was used, equipped with a high brightness Schottky field emitter for low noise imaging fast scanning rates. The SEM is fitted with an In-Beam SE detector. An accelerating voltage of 15 kV and beam currents of about 1 nA gives satisfactory results regarding maximum throughput. Magnification 40 KX was used.

### Feed ration analysis

All feed samples were oven-dried at a temperature of up to 50 °C, then ground with a grinder to a particle size of 1 mm and analyzed for the basic nutrient content. The fiber was analyzed on an A200 Fiber Analyzer (ANKOM, Czech Republic), nitrogenous substances according to the Kjeldahl method (N × 6.25), fat (by direct extraction according to Soxhlet method). The ash was analyzed using a calorimeter (IKA C 5000 Werke, Germany) after burning for 4.5 h in an oven at 550 °C. Energy (combustion heat, BE).

### Amino acid analysis (AAA)

The method for AAA was adopted from Husek and Sweeley et al. In brief, 5 g of hydrolyzed sample was mixed with 25 mL of acid water. The extracts were filtered and passed through an SCX cartridge, previously conditioned according to manufacturer protocol (UCT, Bristol, USA). The obtained solution was dried under N2. Each dried residue was dissolved in 60 µL water and 40 µL ethanol/pyridine (4:1). Five µL ethyl chloroformate was added to the mixture. Finally, 150 µL of chloroform was added.

Derivatized samples were analyzed using the GC system (Agilent 6890, Santa Clara, CA) equipped with an FID. Separation of compounds was conducted on a 10 m CP-Sil 19 capillary column (Agilent, USA) using nitrogen 5.0 as the carrier gas (Siad, Czech republic). The injection volume was 1 µL, and the flow rate was set at 0.7 mL/min. The injector temperature was 250 °C with a split ratio of 50:1, and the FID temperature was 250 °C. The oven temperature was programmed as follows: the column was held initially at 140 °C, then increased to 280 °C at 40 °C/min and held for 3 min. Chromatographic data were recorded and integrated using Clarity software (Data Apex, Czech republic) [28].

### Blood analysis

Blood samples were collected from all experimental animals to determine haematological (haemoglobin, hematocrit, erythrocytes, leukocytes, and platelets) and biochemical parameters (albumin, AST, GGT, glucose, total bilirubin and protein, urea). Spectrometric analyses were performed by a Konelab T20xt biochemical analyzer (Thermo Fisher Scientific) and commercially available reagents, according to [29]. Furthermore, glutathione peroxidase (GPx) and malondialdehyde (MDA) were monitored in the blood according to Urbankova et al. [30]. Blood was collected from the external jugular vein into plastic containers with heparin as an anticoagulant.

Colorimetric assay of determination of MDA and GSH / GSSG was carried out according to the manufacturer protocol (Elabscience, USA).

### Histopathology analysis

From each slaughtered pig, the entire liver and kidneys were removed and weighed, then the mass of these organs was converted to the percentage of the live animal weight. Tissue samples of the liver (taken from the right lobe of the liver, lobus hepatis dexter) and the intestine (taken from the middle jejunum, 3 cm) were collected and immediately fixed in 10% formaldehyde solution to investigate and evaluate pathomorphological changes. Fragments of tissues were cut at 3.0 μm, then positioned onto Superfrost Plus slides (Leica, UK) with the orientation core placed up on the slide. All tissue blocks were oriented the same way; then, the entire tissue block was cut with the remaining sections dipped in wax and stored at room temperature. The sections were stained with hematoxylin and eosin according to standard procedures. Pictures were taken using an inverted Olympus microscope IX 71S8F-3 (Tokyo, Japan) at the magnification 10–20 × for liver samples and 10 × magnification for jejunum.

### Statistics

The data were analyzed using STATISTICA.CZ, version 12.0 (Czech Republic). The results were expressed as a mean from all samples ± standard deviation (n = 3). Statistical significance was determined by examining the basic differences among groups using ANOVA and Schaffer's method for all parameters. The differences with *p* < 0.05 were considered significant.

## Data Availability

The datasets used and/or analyzed during the current study are available from the corresponding author on reasonable request.

## Abbreviations

AST: Aspartate transaminase
C: Control group of pigs
CREA: Creatinine
GGT: Gamma-glutamyl transferase
GLUC: Glucose
GPx: Glutathione peroxidase
GSH: Reduced glutathione
GSSG: Oxidized glutathione
HPF: High-power field
MDA: Malondialdehyde
SDH: Sorbitol dehydrogenase
T: Treated group of pigs
