# Correction to: Is a new generation of mycotoxin clay adsorbents safe in a pig’s diet?

**DOI:** 10.1186/s40813-022-00276-9

**Published:** 2022-07-25

**Authors:** Pavel Horky, Pavel Nevrkla, Tomas Kopec, Iqra Bano, Misa Skoric, Jiri Skladanka, Sylvie Skalickova

**Affiliations:** 1 Department of Animal Nutrition and Forage Production, Faculty of AgriSciences, Mendel University in Brno, Zemedelska 1, 613 00 Brno, Czech Republic; 2 Department of Animal Breeding, Faculty of AgriSciences, Mendel University in Brno, Zemedelska 1, 613 00 Brno, Czech Republic; 3 Department of Pathological Morphology and Parasitology, Faculty of Veterinary Medicine, University of Veterinary and Pharmaceutical Sciences Brno, Palackeho trida 1946/1, Brno, 61200 Czech Republic; 4 Department of Physiology and Biochemistry, Faculty of Bio-Sciences, Shaheed Benazir Bhutto University of Veterinary and Animal Sciences, Sakrand, 67210 Sindh Pakistan

## Correction to: Porcine Health Management 8:31 (2022) 10.1186/s40813-022-00275-w

Following publication of the original article [1] it was reported that Pavel Nevrkla’s family name was misspelled ‘Nevrka’. The correct author list is given in this Correction article and the original article [1] has been updated.
